# No evidence for increased habitual or decreased goal-directed action control after acute stress

**DOI:** 10.1371/journal.pone.0327807

**Published:** 2025-09-08

**Authors:** Katharina Zwosta, Tatjana Karcz, Marcus Möschl, Moritz Walser, Thomas Goschke, Hannes Ruge, Uta Wolfensteller

**Affiliations:** Department of Psychology, Technische Universität Dresden, Dresden, Germany; University of Porto Faculty of Psychology and Educational Sciences: Universidade do Porto Faculdade de Psicologia e de Ciencias da Educacao, PORTUGAL

## Abstract

Previous studies suggested that acute stress can impair flexible goal-directed action control in favor of habitual action control. In addition, there is evidence that acute stress differentially affects the processing of rewards and punishments. Therefore, we aimed at investigating whether acute stress affects the balance between goal-directed and habitual behavior not only for behavior aiming at reward but also for behavior motivated by avoiding punishments. In two experiments, a total of 129 participants either underwent a standardized procedure to induce acute stress or a control procedure. Habitual approach and avoidance behavior was established by extensively training participants on responses resulting in either gaining a monetary reward or avoiding a monetary loss. Subsequently, the strength of the resulting habits was tested in the habit-goal competition task. We found no evidence for acute stress to influence the acquisition of novel behaviors or the resulting habit strength, neither for approach nor for avoidance behavior. This result remained when inter-individual differences in cortisol reactivity and subjective reports of chronic stress were taken into account. Together, our results speak against a general effect of acute stress boosting the behavioral impact of habitual behavior on goal-directed action control.

## Introduction

Human behavior can be more or less controlled by anticipated consequences. While goal-directed behavior involves action selection according to desired outcomes, habitual behavior is assumed to be controlled by stimuli evoking particular responses [1]. Previous research suggested that acute stress can shift behavioral control from a goal-directed to a more habitual mode. Seminal studies investigating the impact of acute stress on goal-directed and habitual behavior employed a devaluation paradigm in which subjects learn that specific actions in the presence of specific stimuli lead to different rewarding outcomes [2,3]. Subsequently, one of the outcomes is devalued and it is tested whether subjects still perform the formerly rewarded action. The authors reported that subjects who experienced acute stress continued to show previously rewarded behavior even when the outcome was no longer desired, both when they were stressed before learning [2] as well as when the stress induction procedure was performed after outcome devaluation [3]. However, a recent study failed to replicate the original findings showing no difference between stressed and non-stressed participants in goal-directed behavior after devaluation [4].

Additional studies which employed different paradigms to study goal-directed and habitual behavior, such as the slips-of-actions task [5,6], the two-step Markov task [7–9] and a different devaluation paradigm [10] mostly indicate that the induction of acute stress alone does not necessarily shift the balance between goal-directed and habitual behavior. For instance, in one study a larger rate of habitual responding was found only in participants who displayed a substantial increase in salivary cortisol after the stress induction procedure [6]. Interestingly, Radenbach and colleagues [9] who manipulated acute stress within subjects found that cortisol reactivity after acute stress was related to the balance between goal-directed and habitual behavior both after acute stress but also after a non-stress-inducing control procedure, suggesting that cortisol reactivity as a trait might be related to goal-directed and habitual behavior. In addition, they found that acute stress only affected the balance between goal-directed and habitual behavior in interaction with chronic stress. Another study reported that the cortisol response was negatively correlated with the behavioral contribution of model-based, i.e., goal-directed, computations, but only in participants with low working memory capacity [7]. The notion that impaired goal-directed behavior depends on interindividual differences in working memory capacity was also confirmed by Quaedflieg and colleagues [10] who reported increased habitual responding only for participants with low working memory capacity. Finally, even when similar tasks were used, Otto and colleagues [7] reported stress effects on model-based behavior while Park and colleagues [8] reported that acute stress increased model-free behavior but only after trials where a possible reward was absent.

Together, these results draw a rather intricate picture of the effects of acute stress, which goes beyond a simple connection between acute stress and increased habitual behavior. Instead, behavioral effects might depend on additional moderators, such as cortisol reactivity, chronic stress as well as cognitive ability, which might also be of variable importance for different tasks. What all these studies to date have in common is that they investigated the goal-directedness of behavior aiming at reward. The research on stress effects on the goal-directedness of avoidance behavior is scarcer. Interestingly, acute stress has been found to exert differential effects on learning from reward and from punishment [11,12]. In particular, stress was found to augment the behavioral impact and neural processing of reward [13,14] and to reduce learning from punishment [12,13]. Studies on flexible avoidance reversal learning have revealed differing results, one study reported impaired reversal learning and increased avoidance habits [15] while another reported unimpaired avoidance reversal learning following acute stress [16]. Taken together, the experimental procedures used to study the balance between goal-directed and habitual behavior in approach and avoidance behavior so far are quite varied. Moreover, differing stress protocols used to induce acute stress constitute another potential source of variance.

The aim of the present study was to investigate effects of acute stress on goal-directed and habitual tendencies in both approach and avoidance behavior using the same experimental paradigm, the habit-goal competition task [17]. In this paradigm approach and avoidance behavior is extensively trained and then, in a test phase, put into competition with goal-directed behavior. Habit formation is measured by comparing goal-habit compatible trials, where the required goal-directed response is identical to the trained response, with incompatible trials, where both responses are different. This paradigm offers indicators for habit formation both in terms of response selection, when the trained response is selected instead of the required goal-directed response, and in terms of response time slowing, when the goal-directed response is selected but response times are slower due to interference with the competing trained response. Such response times effects have been argued to be a more sensitive indicator for habit formation than overt response selection [18].

We conducted two experiments in which we used two different standardized protocols, to induce psychosocial stress, the Trier Social Stress Test (TSST) [19] or a combination of physical and psychosocial stress, the Maastricht Acute Stress Test (MAST) [20], in half of the participants and control procedures in the other half. We then trained participants by either contingently rewarding (thus inducing approach habits) or punishing one particular response (thus inducing avoidance habits) for each stimulus. After removing the contingency between the responses and the outcomes, we tested the effect of acute stress on goal-directed and habitual behavior by examining the behavioral interference of the previously trained actions with goal-directed behavior and free-choice behavior [17]. In order to maximize statistical power, both experiments were analyzed jointly. If acute stress indeed promotes a shift toward habitual control, we would expect greater behavioral interference effects, reflected in larger compatibility effects, for response times and error rates as well as a stronger tendency to select the previously trained response during free-choice trials. Additionally, we explored whether these effects differed between approach and avoidance habits.

## Methods

### Subjects

All participants provided written informed consent. The experiments were approved by the ethics committee at TU Dresden (EK 415092015 and EK 545122015). The recruitment period for Experiment 1 started on March 16, 2016 and ended on July 29, 2016. Recruitment for Experiment 2 started on January 30, 2018 and ended on June 12, 2018.

Potential participants were screened beforehand and were included in the sample only if they were between 18 and 30 years old, non-smokers, did not use oral contraceptives or other medications influencing cortisol levels, had a body mass index between 16 and 27 kg/m^2^, reported no current or chronic illness and did not partake in excessive sport activities. In addition, to minimize noise in the behavioral data, subjects had to be right-handed and to have unimpaired color vision.

The total sample consisted of 152 subjects. Fifteen subjects were excluded because they did not reach at least 65% correct trials during the learning phase in either approach or avoidance trials. An additional seven subjects were excluded because they did not reach at least 65% correct trials in goal-directed trials with no interfering habits. One subject was excluded because they had already performed the experimental paradigm in a previous study. The final sample comprised 129 subjects of which 64 subjects participated in Experiment 1 (35 male, 29 female) and 65 subjects in Experiment 2 (34 male, 31 female). At the time of the experiment, their mean age was 23.4 years (*SD* = 3.0 years) and their mean BMI was 22.1 kg/m^2^ (*SD* = 2.2 kg/m^2^).

Power analyses were conducted with G*Power 3 [21] to determine the achieved power with this sample size to detect small or medium sized effects. Note that, while these analyses were conducted after data collection, they still follow the logic of a-priori power analyses. This revealed a power of more than 99% with our sample size to detect a significant interaction effect (between compatibility and stress group) in a mixed-ANOVA with a medium effect size (partial η^2 ^= 0.06). For a small effect size (partial η^2 ^= 0.01), the achieved power is 89% for error rates (correlation among repeated measures: *r* = .744) and more than 99% for response times (correlation among repeated measures: *r* = .956).

The chronic stress level of participants was assessed using the Perceived Stress Scale (PSS-10) [22,23] and the Trier Inventory for Chronic Stress (TICS) [24]. In addition, we assessed trait impulsiveness using the Barratt Impulsiveness Scale (BIS-11) [25,26] and the need for cognition using the Need for Cognition Scale (NFC) [27]. Descriptives regarding these measures are provided in Table 1.

**Table 1:** Descriptive Statistics of Questionnaire Scores.

	Experiment 1 (TSST)		Experiment 2 (MAST)	
	Stress N = 31	No-Stress N = 33	Stress N = 32	No-Stress N = 33
PSS-10	24.4 (4.9)	23.8 (5.3)	23.3 (4.3)	22.9 (4.8)
TICS	53.5 (12.6)	54.7 (19.5)	53.9 (19.9)	51.3 (13.1)
BIS-11	60.8 (9.3)	60.6 (10.1)	57.6 (9.3)	60.3 (11.9)
NFC	59.1 (7.5)	60.1 (8.2)	60.0 (6.8)	60.0 (6.8)

Mean scores are given with SD in parentheses. Scores did not differ between stress groups or stress induction methods. PSS-10: Perceived Stress Scale; TICS: Trier Inventory for Chronic Stress; BIS-11: Barratt Impulsiveness Scale; NFC: Need for Cognition Scale.

### Experimental procedure

In order to achieve comparable blood glucose levels across participants and to facilitate a robust cortisol response to the stress induction procedure [28,29] they were instructed to not consume any food during the two hours before the experiment. Upon their arrival, they were then asked to drink a glass of grape juice in line with recommendations for the TSST [30,31]. While glucose can affect cognitive processes, particularly memory [32,33], any effects of glucose should affect both groups equally and therefore not confound the comparison between stress and control conditions.

Both experiments were identical in their procedures except for the stress induction method, which was either the TSST [19] or the MAST [20], thus allowing us to assess the impact of stress on habitual and goal-directed behavior across different stress induction methods. In each of the experiments acute stress was manipulated between subjects with one half of the subjects undergoing the standard version of the stress induction procedure and the other half undergoing a placebo version which is similar to the original version but does not elicit a stress response [34].

**Fig 1:**
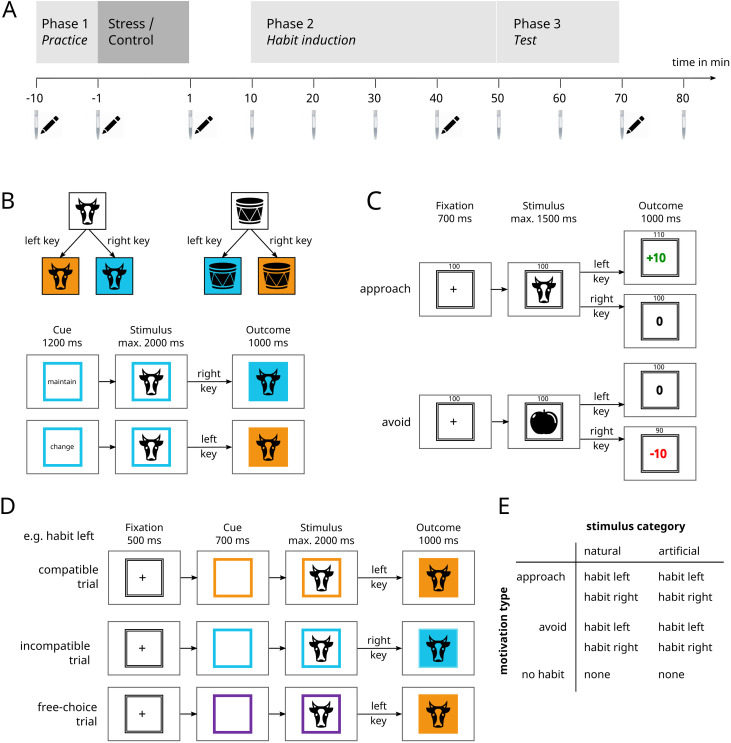
Experimental Procedure. **A.** Timeline of the experiment. Time is indicated in reference to the stress induction/ control procedure which lasted approximately 15 minutes. The time points of saliva samples are illustrated by saliva sampling devices and subjective mood reports by icons of pens. **B.** Examples of the instructed stimulus-response outcome associations and trials in Phase 1 (goal-directed practice). **C.** Exemplary trial procedures in Phase 2 (habit induction). **D.** Examples for compatible, incompatible and free-choice trials in Phase 3 (test phase – goal-habit competition). **E.** Table depicting the counterbalancing of stimulus category, motivation type, and trained response. Each cell represents one of the ten stimuli.

The experimental paradigm consists of three consecutive phases [17] and the stress induction procedure was performed after the first practice phase (see Fig 1A). A detailed description of the experimental paradigm, including the practice phase, can be found in [17]. In the practice phase subjects learned the relationship between two categories of stimuli, two response options and resulting color outcomes. This enabled them to intentionally produce a specific color outcome in the presence of a particular stimulus by choosing their response accordingly. After this stage, the stress induction procedure or the placebo version of the procedure was performed. After a delay of 10 minutes, habits were induced by contingently either rewarding one particular response towards a stimulus or allowing participants to avoid punishment by executing a response. Then finally, the presence of habits was tested by having subjects again perform according to the goal-directed contingencies acquired in the first phase while the responses trained in the second phase could be either in accordance or in conflict with the goal-directed responses.

#### Phase 1: Goal-directed practice phase.

In the first part of the experiment, two categories of stimuli, five “artificial” and five “natural” stimuli, were used (natural stimuli: tree, snowflake, cow, mushroom, lungs; artificial stimuli: scissors, computer mouse, car, cupboard, ball). The stimuli were black-and-white, vertically symmetrical icons of different objects. Responses were made with the keyboard, pressing the key “D” with the left index finger or the key “K” with the right index finger. Responding to a stimulus from one group of stimuli (e.g., artificial) with the right key led to a blue outcome and responding with the left key led to an orange outcome. This response-outcome association was inverted for the other group of stimuli (e.g., natural), such that pressing the right key led to an orange outcome and pressing the left key led to a blue outcome (see Fig 1B).

The first phase consisted of a total of 240 trials. Each trial started with the presentation of a cue containing either the German words for “change” or “maintain”. This word was framed by a colored square displaying the present outcome color that was produced in the previous trial (or a random color in the first trial). After 1200 ms the cue was replaced by one of the ten stimuli. The participants’ task was now to press the key that would either change or maintain the current outcome color for the group the displayed stimulus belonged to. The response window was set to 2000 ms. As soon as the response was made, the background of the stimulus turned to the respective outcome color for 1000 ms.

#### Stress induction phase.

After phase 1 the stress induction procedure was performed. Half of the subjects underwent the standard version of the TSST or MAST which aims at inducing a subjective and physiological stress response. The other half of the participants underwent the respective control procedure.

#### Phase 2: Habit induction phase.

After a delay of 10 minutes, the second phase of the experiment aimed at inducing habits either by rewarding one of the two responses or by punishing the alternative response, leading to habitual approach or avoidance behavior respectively (see Fig 1C). Eight of the ten stimuli (four artificial and four natural stimuli) were re-used from phase 1. At the beginning, subjects were instructed that the categories of the stimuli were irrelevant for the next task but that they had to find out the correct key for each of the eight stimuli individually by trial-and-error. For both stimulus categories, each of the four stimuli belonging to one category was associated with one of the four combinations of correct responses (left or right) and outcome types (reward or punishment avoidance). They were also told that for four of the stimuli the correct response would allow them to gain points while for the other four stimuli the correct response would allow them to avoid losing points. The respective alternative response would not lead to a change in points. The points would be worth actual money, which they would receive at the end of the experiment. They would also gain additional money depending on their response speed. Participants received one cent per ten points and additionally one cent per ms of their average response time being below 500 ms.

Each trial started with a fixation cross framed by a black-and-white lined square which was displayed for 500 ms. Afterwards one of the stimuli appeared for a maximum of 1500 ms. As soon as the response was made, subjects were shown the point outcome. Rewards were printed in green color and were always “+10”, punishments were printed in red and were “-10” and null outcomes were printed in black and indicated by “0”. If participants failed to execute any response during the response window, they also received the unfavorable outcome of −10 points for avoidance trials and 0 points for approach trials. The outcome stayed on screen for 1000 ms. Throughout a trial the number of currently collected points during the present task block was displayed at the top of the screen.

Phase 2 consisted of 784 trials (98 per stimulus). Every 8.5 min the experiment was interrupted at the end of the current trial, the total points collected during this block and the mean response time for correct responses were displayed and a saliva sample was collected by the experimenter. The experiment was then resumed 1.5 min after it was stopped. Points were converted into money at the end of the experiment.

#### Phase 3: Test phase – goal-habit competition.

In phase 3 the goal-directed behavior established in phase 1 was placed into competition with the habitual behavior induced during phase 2 in order to test the individual habit strength (see Fig 1D). At the beginning of the third experimental phase we instructed subjects that they could no longer gain or lose any points and thereby removed the contingency between stimulus, response and monetary outcome. This instruction-based approach allowed us to remove rewards and punishments in a comparable manner. Hence, any tendency to continue to perform the trained response established in phase 2 should not be motivated by aiming to gain reward or avoid loss but should be based on habitual responding instead.

Further, the contingencies, introduced in phase 1, between stimuli, responses and outcome colors were reinstructed, again containing all ten stimuli. Afterwards a display showed the task instructions for the third phase: Each trial started with a fixation cross, followed by a colored frame (cue) which was either one of the two outcome colors (blue and orange) previously introduced in phase 1, or a new third color (purple) indicating free-choice trials. Afterwards, one of the ten stimuli appeared in the center of the frame. If the frame was blue or orange, subjects were required to press the response that would lead to this particular outcome color for the displayed stimulus according to the S-R-O contingencies from phase 1 (goal-directed trials). If, however, the frame was purple then subjects could freely choose one of the two responses (free-choice trials). In order to prevent response selection prior to stimulus presentation, 6% of the trials were catch trials, in which the purple cue was followed by an arrow pointing to left or right, in response to which subjects were supposed to press the key in the corresponding location. Additionally, they were explicitly told not to decide for their response before the stimulus appeared.

Independent of trial type, the fixation cross was displayed for 500 ms followed by the cue frame for 700 ms. The stimulus then appeared for a maximum of 2000 ms or until a response was made and finally the outcome color was shown for 1000 ms.

The whole phase 3 comprised 384 trials. Apart from the 24 arrow catch trials, there were eight different trial types of interest which appeared in randomized order: Within goal-directed trials there were trials for which the habitual response towards the stimulus was identical to the required goal-directed response either because it had previously been rewarded (*approach compatible*, 48 trials) or not punished (*avoid compatible*, 48 trials). Analogously, in other trials the habitual response did not match the goal-directed response (*approach incompatible* or *avoid incompatible*, each 48 trials). Furthermore, there were goal-directed trials where one of the two stimuli appeared which did not appear in phase 2 and hence for which no habitual response existed (*no habit, goal-only*, 48 trials). Finally, free-choice trials also employed stimuli for which a habitual response was induced in phase 2 (a*pproach free-choice* or *avoid free-choice*, each 48 trials) and stimuli that did not appear in the habit induction phase (*no habit free-choice*, 24 trials).

As during phase 2, the experiment was interrupted every 8.5 min at the end of the current trial and a saliva sample was collected by the experimenter. The experiment was then resumed 1.5 min after it was stopped.

### Stress induction

#### Experiment 1: Trier Social Stress Test.

The TSST is a standardized procedure to induce acute psychosocial stress [19]. Participants were asked to perform a fictional job interview and to solve an arithmetic task in the presence of a jury, who is trained to behave neutrally. The jury consisted of two people, which did not include the experimenter and always included one woman and one man. The participant was positioned in front of a microphone and a camera and was told that they were being recorded and that the members of the jury were trained to analyze the participant’s non-verbal behavior. The control version of the TSST [34] required the subject to talk in an empty room without an evaluating jury. Both versions of the TSST lasted 15 minutes. Half of the experiments were conducted in the morning (starting at 10 am) and the other half was conducted in the afternoon (starting at 1 pm). For both the stress and the control groups, the participants’ gender and the time of the experiment were counterbalanced.

#### Experiment 2: Maastricht Acute Stress Test.

The MAST combines socio-evaluative stress with physiological stress [20]. Participants were asked to put their foot into a tub with ice-cold water (2°C) and to perform an arithmetic task while their facial expression was recorded on camera and they were told that it would be analyzed. Note that in the original version of the MAST the participants hold their hand into cold water [20]. However, in order to avoid any interference of this intervention with the manual responses during the subsequent task, we had participants put their foot into the water instead (see also, e.g., [35]). In contrast to the TSST, there was no designated jury present but only the experimenter. Therefore, the gender of the experimenter was counterbalanced across subjects, such that men and women were equally often tested by male and female experimenters both in the stress and the control groups. In the control procedure the temperature of the water was lukewarm (35°C) and instead of a difficult arithmetic task subjects were asked to perform a simple counting task. The experimenter was present but did not give feedback and the participants were not recorded. Like the TSST, the MAST and the control procedure each had a total duration of 15 minutes.

### Measurement of objective and subjective indicators of the stress response

In order to obtain physiological indicators of the stress response saliva samples were collected using Salivette sampling devices which were analyzed regarding levels of salivary α-amylase (sAA), reflecting activity of the sympathetic nervous system, and free cortisol as an indicator of activity of the HPA axis. For each subject 11 saliva samples were collected. Two samples were collected prior to stress induction: The first sample was collected before subjects started phase 1 and the second sample after subjects completed phase 1 before they underwent the stress induction procedure. After stress induction, nine samples were collected in intervals of ten minutes.

In order to obtain indicators of subjective stress the German version of the multidimensional mood state questionnaire (Multidimensionaler Befindlichkeitsfragebogen, MDBF) [36] was used which measures mood in respect to valence (scale good mood: good – bad), arousal (scale calmness: calm – nervous) and alertness (awake-tired). The MDBF was administered together with the saliva samples at the two time points before stress induction as well as at the time points of 1, 10, 40 and 70 minutes after stress induction.

## Results

### Objective and subjective indicators of the stress response

#### Salivary cortisol and α-amylase.

The data were analyzed for those subjects for whom the complete time series of samples was available. Those were 124 of the 129 participants for salivary cortisol and 116 participants for salivary α-amylase, as some samples did not contain enough saliva to analyze both. ANOVAs were calculated with the within-subject factor time point [1–11] and the between-subject factors stress group (stress vs. control) and experiment (TSST vs. MAST).

**Fig 2:**
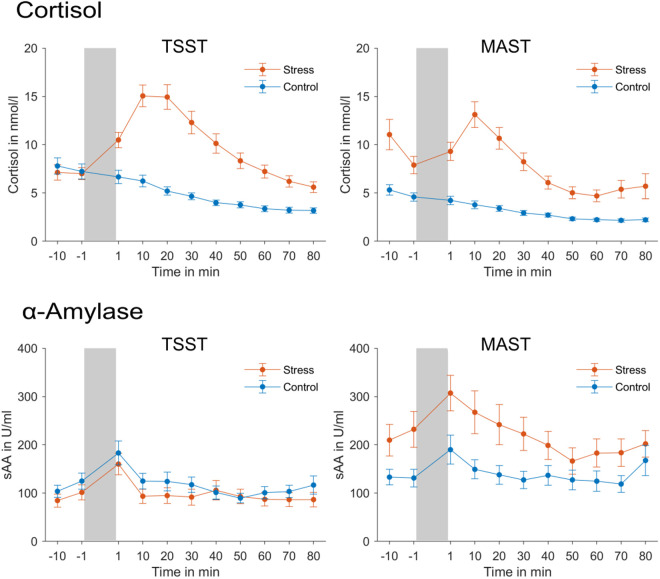
Mean salivary cortisol and α-amylase levels across the experiments. The grey bars denote the time of the stress induction/ control procedures. Error bars represent standard errors of the mean.

Cortisol values significantly changed over time, *F*(10, 1200) = 54.92, *p* < .001, η^2^ = .31. As expected, salivary cortisol levels were higher for subjects who underwent the stress induction procedures compared to subjects who underwent the respective control procedures (see Fig 2), *F*(1, 120) = 66.44, *p* < .001, η^2^ = .36. Importantly, there was also a significant interaction between time point and stress group, *F*(10, 1200) = 23.62, *p* < .001, η^2^ = .16, as there was a sharp increase of cortisol levels after stress induction in the stress group and a slow decrease of cortisol levels across the experiment in the control group. Furthermore, cortisol levels differed between the two experiments, *F*(1, 120) = 8.89, *p* = .003, η^2^ = .07, and changes in cortisol over time were different for the two experiments, *F*(10, 1200) = 5.31, *p* < .001, η^2^ = .04. There was also a significant three-way interaction between time point, stress group and experiment, *F*(10, 1200) = 7.90, *p* < .001, η^2^ = .06. Post-hoc analyses separate for the two experiments revealed that in the TSST experiment, cortisol levels did not differ between the stress groups before stress induction, *p* = .580 and *p* = .787, but were significantly higher in participants who underwent the stress induction procedure compared to the control procedure at all time points after stress induction, all *p*s < .001. By contrast, in the MAST experiment, the stress groups significantly differed in their cortisol levels at all time points, even before the stress induction procedure, all *p*s < .01. Elevated cortisol levels prior to stress induction via MAST have been previously reported (see, e.g., [37]). Importantly though, the sharp increase in cortisol levels following stress induction was observed in both the TSST and the MAST experiment in the stress group, but not in the control group.

For salivary α-amylase there was only a significant main effect of time point (see Fig 2), *F*(10, 1120) = 9.89, *p* < .001, η^2^ = .08, as sAA levels generally increased after stress induction, but there was no significant interaction between time point and group, *F*(10, 1120) = 0.62, *p* = .795. Comparable effects of TSST stress and control manipulations on α-amylase levels have been previously reported (see, e.g., [34], [38]). Instead, there was a significant main effect of experiment, *F*(1, 12) = 14.23, *p* = .001, η^2^ = .11, with higher sAA levels in the MAST than in the TSST experiment, and a significant interaction between experiment and stress group, *F*(1, 112) = 6.56, *p* = .012, η^2^ = .06. Separate analyses for the two experiments showed that in the MAST experiment sAA levels were overall higher in the stress compared to the control group, *F*(1, 58) = 6.36, *p* = .014, η^2^ = .10, which was not the case in the TSST experiment, *F*(1, 54) =.81, *p* = .372.

#### Subjective mood ratings.

The subjective mood ratings were analyzed for those subjects who completed all items of the respective MDBF scale at all time points. Of the original 129 subjects, 125 subjects remained for the good mood scale, 124 for calmness and 126 for alertness. In order to analyze the ratings, ANOVAs were calculated with the within-subject factor time point (1 to 6) and the between-subject factors stress group (stress vs. control) and experiment (TSST vs. MAST).

**Fig 3:**
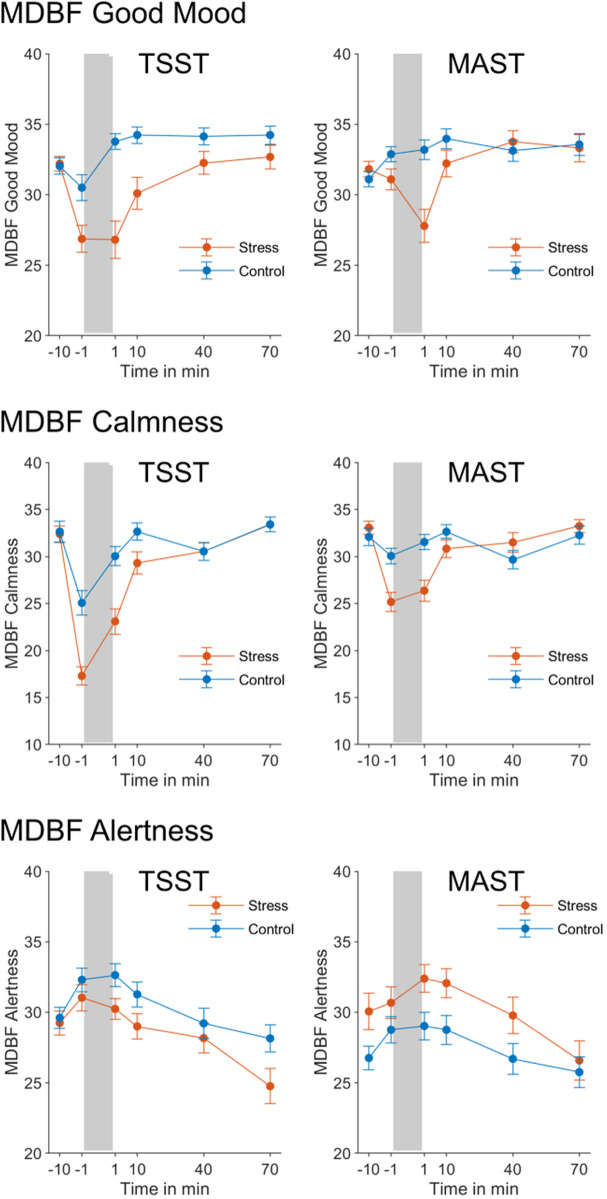
Subjective mood ratings across the experiments. Error bars denote SEMs.

For the subjective ratings of good mood, there were significant main effects of stress group, *F*(1, 121) = 11.25, *p* = .001, η^2^ = .09, as well as time point, *F*(5, 605) = 26.73, *p* < .001, η^2^ = .18. Importantly, there was a significant interaction between time point and stress group, *F*(5, 605) = 19.26, *p* < .001, η^2^ = .14. Good mood ratings dropped for the participants who underwent the stress induction procedure but not for participants who performed the control procedure (see Fig 3, upper panel). In addition, there was a significant interaction between time point and experiment, *F*(5, 605) = 6.67, *p* < .001, η^2^ = .05, as subjective good mood was lower immediately before the TSST/control TSST than before the MAST/ control MAST.

Similarly, for the calmness ratings there were also significant main effects of stress group, *F*(1, 120) = 9.02, *p* = .003, η^2^ = .07, and time point, *F*(5, 600) = 93.10, *p* < .001, η^2^ = .44, as well as a significant interaction effect between stress group and time point, *F*(5, 600) = 23.66, *p* < .001, η^2^ = .17. Calmness ratings dropped more strongly for participants who underwent the stress induction procedure than for participants who underwent the control procedure. In addition, calmness ratings differed between the two experiments, *F*(1, 120) = 4.21, *p* = .042, η^2^ = .03 and showed different changes over time in both experiments, *F*(5, 600) = 15.14, *p* < .001, η^2^ = .11. Again, this effect was driven by calmness ratings dropping more strongly immediately before the stress induction/control procedure in the TSST than in the MAST experiment.

For the alertness scale, there was a significant main effect of time point, *F*(5, 610) = 28.85, *p* < .001, η^2^ = .19. However, there was no significant interaction effect between stress group and time point, *F*(5, 610) = 2.00, *p* = .076. There was only a significant interaction between stress group and experiment, *F*(1, 122) = 7.29, *p* = .008, η^2^ = .06, such that in the TSST experiment alertness was higher in the control group than the stress group while in the MAST experiment this pattern was reversed.

### Effects of stress on goal-directed and habitual behavior

#### Phase 2: Effects of stress on habit acquisition.

ANOVAS were calculated with the within-subject factors block (1 to 7) and motivation (approach vs. avoid) and the between-subject factors stress group (stress vs. control) and experiment (TSST vs. MAST).

**Fig 4:**
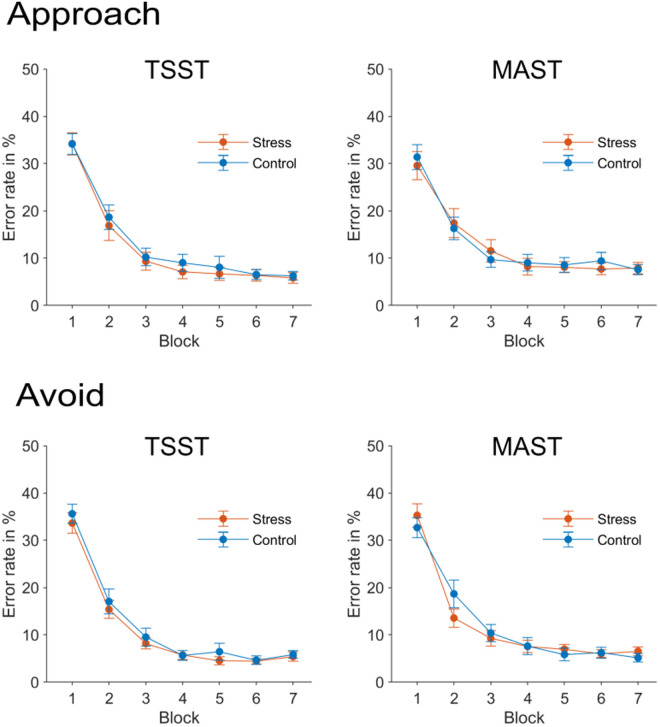
Error rates across the habit induction phase (phase 2). Error bars denote SEMs.

As expected, error rates decreased during the habit induction phase (see Fig 4), *F*(6, 750) = 328.07, *p* < .001, η^2^ = .72. This decrease was slightly stronger for avoidance than for approach trials, as indicated by a significant interaction between the factors block and motivation, *F*(6, 750) = 2.64, *p* < .048, η^2^ = .02. Importantly, there was neither a significant main effect of stress group, *F*(1, 125) = 0.30, *p* = .581, nor an interaction between stress group and block, *F*(6, 726) = 0.34, *p* = .919.

Response times also decreased across the habit induction phase, *F*(6, 750) = 229.44, *p* < .001, η^2^ = .65. Furthermore, subjects were faster when they could gain monetary rewards than when they were avoiding loss, *F*(1, 125) = 190.69, *p* < .001, η^2^ = .60. This difference was more pronounced at the beginning than at the end of the habit induction phase, *F*(6, 750) = 16.79, *p* < .001, η^2^ = .12. However, again, there was neither a significant effect of stress group, *F*(1, 125) = 0.98, *p* = .324, nor of the interaction between stress group and block, *F*(6, 750) = 1.59, *p* = .148.

#### Phase 3: Effects of stress on the resulting habit strength.

In order to test stress effects on habit strength, we analyzed error rates and response times during the test phase. ANOVAs were calculated with the within-subject factors goal-habit compatibility (compatible vs. incompatible) and motivation (approach vs. avoid) and the between-subject factors stress group (stress vs. control) and experiment (TSST vs. MAST). Additional ANOVAs with only the between-subject factors were calculated for the goal-directed trials using stimuli for which no response-habit had been inducted in phase 2.

**Fig 5:**
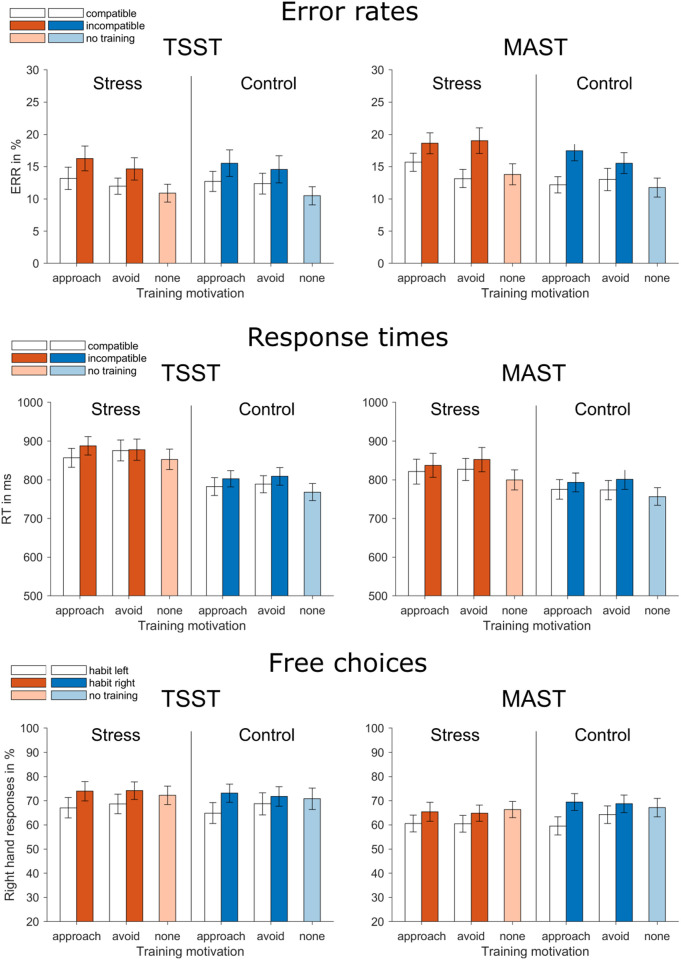
Error rates, response times and free choices in the test phase. Error bars denote SEMs.

Error rates were significantly higher in incompatible than compatible trials, *F*(1, 125) = 34.98, *p* < .001, η^2^ = .22 (see Fig 5). In addition, there was a significant main effect of motivation, *F*(1, 125) = 4.55, *p* = .035, η^2^ = .04, reflecting that error rates were slightly lower for avoid than for approach trials. Importantly, stress did not significantly affect this compatibility effect, *F*(1, 125) = 0.13, *p* = .716, and there were also no three-way interaction between stress group, compatibility and motivation, *F*(1, 125) = 2.36, *p* = .127, and no four-way interaction between stress group, compatibility, motivation and experiment, *F*(1, 125) = 2.01, *p* = .159. Stress also did not significantly influence error rates in trials without an interfering habitual response, *F*(1, 125) = 0.70, *p* = .406.

The analysis of response times revealed that, as expected, subjects were faster in compatible than incompatible trials, *F*(1, 125) = 26.27, *p* < .001, η^2^ = .17 (see Fig 5). Furthermore, there was a significant main effect of stress group such that stressed participants generally showed prolonged response times compared to non-stressed participants, *F*(1, 125) = 6.31, *p* = .013, η^2^ = .05. This was corroborated by a significant main effect of stress group for trials with no trained response, *F*(1, 125) = 6.93, *p* = .010, η^2^ = .05, again reflecting longer response times after stress than after the control procedure. There were no other significant effects. Compatibility effects in response times were not significantly influenced by stress, *F*(1, 125) = 0.15, *p* = .701, and this was also not modulated by motivation, *F*(1, 125) = 1.28, *p* = .261. Again, there was also no significant four-way interaction between stress group, compatibility, motivation and experiment, *F*(1, 125) = 1.11, *p* = .293. In order to clarify whether the main effect of stress group could be interpreted as an effect of the stress manipulation rather than a pre-existing group difference, we compared the mean response times in phase 1 prior to stress induction. The analysis revealed that participants in the stressed participants showed prolonged response times compared to non-stressed participants already prior to stress induction, *t*(126) = 3.21, **p* *= .002, *d* = .57.

In order to analyze whether stress affected response choices in free-choice trials, we calculated the proportions of right-hand responses depending on the induced habit (left vs. right vs. none) and motivation (approach vs. avoid vs. none). Subjects generally preferred the right to the left response in free-choice trials as indicated by a proportion of 69% right hand responses for stimuli with no trained response. In order to determine whether this proportion was influenced by the induced habit and by stress an ANOVA was calculated with the within-subject factors stress group (stress vs. control) and experiment (TSST vs. MAST). This analysis revealed that the proportion of right-hand choices was influenced by the induced habit, *F*(1, 121) = 36.70, *p* < .001, η^2^ = .23. The proportion of right-hand responses was 64% if a left response habit had been induced and 70% if a right response habit had been induced. There was also a marginally significant interaction effect between induced habit and motivation, *F*(1, 125) = 3.96, *p* = .049, η^2^ = .03, which reflects a slightly larger effect of an induced approach habit than an induced avoidance habit on response choices. However, there were no significant interactions between induced habit and stress group, *F*(1, 125) = 0.27, *p* = .601, the three-way interaction between induced habit, stress group and motivation, *F*(1, 125) = 2.02, *p* = .158, and the four-way interaction between induced habit, stress group, motivation and experiment, *F*(1, 125) = 0.02, *p* = .878. Again, a separate ANOVA was calculated for response choices towards stimuli with no associated habits with only the between-subject factors. This analysis revealed no significant stress effect, *F*(1, 125) = 0.01, *p* = .933.

#### Tests of the null hypotheses- no general stress effects on habit strength.

In order to test the null hypotheses that compatibility effects in response times and error rates are generally not influenced by acute stress, we ran a Bayesian *t*-test using the Software JASP [39], comparing the compatibility effects between the stress and the control group with a Cauchy prior distribution with a default scale of .707. Both for the compatibility effect in error rates and response times, the null hypothesis that there is no difference between the groups was five times more likely than the alternative hypothesis that the groups differ, BF_01_ = 4.983 and BF_01_ = 4.969, respectively. We also tested whether the difference in right-hand free-choices between left and right habits was influenced by the stress group. Again, the null hypothesis was five times more likely, BF_01_ = 4.666.

#### Post-hoc analyses: Moderating roles of the cortisol response and chronic stress.

Previous studies have reported that the effects of acute stress were conditional on a substantial cortisol response or scaled with the size of the cortisol response [6,7,9]. Therefore, we also tested for moderating effects of the individual cortisol response. We calculated the maximum cortisol increase for each subject by subtracting the cortisol value immediately before the stress induction/ control procedure (baseline, time point −1 in Figs 1, 3 and 4) from the maximum cortisol value measured 10–40 min afterwards. One subject in the TSST experiment was removed because the baseline cortisol sample was missing. The remaining participants were classified as responders if their maximum cortisol increase was at least 1.5 nmol/l and as non-responders if their maximum increase was below this value [40]. This resulted in 51 responders and 12 non-responders in the stress group (TSST: 27 vs. 4, MAST: 24 vs. 8) and 7 responders vs. 58 non-responders in the control group (TSST: 4 vs. 28, MAST: 3 vs. 30). In order to test whether stress had an effect on habitual behavior when it was accompanied by a substantial cortisol response, we repeated the analyses only for those participants who were in the stress group and classified as responders and those who were in the control group and classified as non-responders.

Both for the habit induction and the test phase, the results were qualitatively replicated. In the habit induction phase, error rates were unaffected by the stress group, *F*(1, 105) = 0.02, *p* = .891, and there were no significant interactions between stress group and block, *F*(6, 630) = 0.21, *p* = .875, stress group, block and motivation, *F*(6, 630) = 0.87, *p* = .459, or stress group, block, motivation and experiment, *F*(6, 630) = 1.58, *p* = .196. Likewise, response times were unaffected by the stress group, *F*(1, 105) = 1.43, *p* = .235, the interaction between block and stress group, *F*(6, 630) = 0.90, *p* = .421, the three-way interaction between stress group, block and motivation, *F*(6, 630) = 0.17, *p* = .924, and the four-way interaction between stress group, block, motivation and experiment, *F*(6, 630) = 1.25, *p* = .293.

For error rates in the test phase, there were no significant interactions between stress and compatibility, *F*(1, 105) = 0.14, *p* = .705, between stress, compatibility and motivation, *F*(1, 105) = 3.09, *p* = .082, between stress, compatibility and experiment, *F*(1, 105) = 0.04, *p* = .840, and between stress, compatibility, motivation and experiment, *F*(1, 105) = 1.06, *p* = .305. Similarly, for response times, there were no significant interactions between stress and compatibility, *F*(1, 105) < 0.01, *p* = .990, between stress, compatibility and experiment, *F*(1, 105) = 0.06, *p* = .815, as well as between stress, compatibility and motivation, *F*(1, 105) = 0.33, *p* = .565, and between stress, compatibility, motivation and experiment, *F*(1, 105) = 0.01, *p* = .970. Again, and even more pronounced than before, response times were overall significantly prolonged in the stress compared with the control group, *F*(1, 105) = 8.28, *p* = .005, η^2^ = .07. However, response times were also already longer in the stress compared with the control group during goal-directed training in phase 1 prior to stress induction, *t*(106) = 3.58, **p* *= .001.

For free-choice trials, there was again only a main effect of induced habit, *F*(1, 106) = 30.72, *p* = < .001, η^2^ = .23, reflecting that participants chose the right-hand response more frequently if this response was trained than if the other response was trained. No other effect was significant, including the interaction between trained response and stress group, *F*(1, 105) = 0.02, *p* = .880.

We also tested for correlations between the behavioral effects and the maximum cortisol increase. To this end, we calculated compatibility effects in response times and error rates by subtracting the values in compatible trials from the values in incompatible trials as well as free-choice biases by subtracting the proportion of right-hand responses for stimuli with trained left-hand responses from the proportion for stimuli with trained right-hand responses. The analyses revealed no significant correlations, neither for error rates, *r* = .025, *p* = .778, nor for response times, *r* = −.052, *p* = .561, and free-choice biases, *r* = .048, *p* = .594. This was also the case when compatibility effects and free-choice biases were calculated separately for approach and avoidance trials, all *p*s > .27.

Finally, it has been reported that the amount of chronic stress influences acute stress effects on goal-directed behavior with more chronic stress leading to more habitual behavior after acute stress [9]. Therefore, we tested for correlations between compatibility effects in response times, error rates and free-choice biases with the questionnaire scores of the Perceived Stress Scale (PSS-10) and the Trier Inventory for Chronic Stress (TICS). These analyses revealed no significant correlations between these questionnaire scores and the behavioral effects, neither in the whole group nor when the analyses were performed only for the stress group, all uncorrected *p*s > .05. There were also no significant correlations when the behavioral effects were calculated separately for approach and avoidance trials, all uncorrected *p*s > .10.

## Discussion

The aim of this study was to investigate effects of acute stress on the balance between goal-directed and habitual approach and avoidance behavior. We conducted two experiments, in which we induced stress with two different stress induction protocols before participants were tested in the habit-goal competition task [17], which allows to measure the behavioral impact of habitual action tendencies on goal-directed behavior in terms of both error rates and response times. Participants were trained to respond to specific stimuli with specific responses either in order to gain reward or to avoid loss. Subsequently, we tested whether and how strongly behavior had become habitual in situations where the trained response tendencies were in competition with goal-directed behavior and in situations where subjects could freely choose a response [17].

We did not find evidence for acute stress to promote habitual behavior. More specifically, both stressed and control groups showed comparable substantial goal-habit interference effects both in terms of error rates and response times for goal-directed responses that were in competition with previously trained (i.e., habitual) responses acquired via reward approach or punishment avoidance motivation. Similarly, the bias towards the previously trained response in free-choice behavior was also unaffected by acute stress. The only difference between the experimental groups was that stressed participants showed prolonged response times in the test phase, independent of whether goal-directed behavior was in accordance or in opposition to habitual response tendencies or whether there was no habitual tendency at all. However, in light of the finding that the response time difference between the groups was present already prior to stress induction, the most parsimonious explanation is a pre-existing difference between the groups that was unrelated to the actual stress induction. We cannot rule out that participants were having slightly different anticipations of the experiment, which might in turn have led to an increased level of cautiousness already prior to stress induction. Yet, in any case this could only be interpreted as a rather unspecific effect, which was apparently not specifically affecting the competition between goal-directed and habitual action control investigated here. Apart from the lack of stress effects on habit strength, we also found that acute stress did not affect the acquisition of approach and avoidance behavior during the learning phase, neither by influencing how rapidly the behavior was learned nor how quickly it was performed.

At first glance our findings appear to be at odds with several previous studies showing that acute stress shifted the balance between goal-directed and habitual behavior in the direction of habitual behavior [2,3,6,8–10]. However, those previous results are far from unequivocal. For instance, the increase in habitual responding reported by Schwabe and Wolf [2,3] was restricted to the very first trials following training, when participants had not realized that actions were no longer followed by (de)valued outcomes. A recent conceptual replication attempt using a stronger devaluation method revealed no evidence for increased habitual control following acute stress [4]. Furthermore, two recent exact replication attempts also revealed no differences between the stress and control groups but failed to demonstrate goal-directed behavior after devaluation even in the control group, which precludes the investigation of a potential stress-induced shift from goal-directed toward habitual behavior by comparing stress and control groups [41]. Other recent studies using different experimental paradigms have similarly failed to produce reliable stress-related shifts towards more habitual behavior. Van Timmeren et al. [42] reported no stress-induced impairments in model-based (goal-directed) learning using a 2-step Markov decision task in a healthy albeit comparatively small sample. In two recent studies using the slips-of-action task, stress did not generally [6] or not reliably interact with action value [43].

Previously, several boundary conditions have been discussed with respect to the influence of acute stress. We will address how those apply to our results. Several studies reported increased habitual behavior to stressed participants classified as cortisol responders [6] or cortisol responders irrespective of experimental stress induction [9]. Here, even when we considered individual cortisol responses by restricting the analysis to participants with substantial cortisol responses after the stress induction procedure or by correlating behavioral effects with the size of the cortisol response, we did not find evidence for acute stress generally promoting habitual behavior. Similarly, we did not find a correlation between subjective chronic stress and a decrease in goal-directed behavior as reported by Radenbach et al. [9]. Some studies have reported stress-related increases in habitual behavior for individuals with low working memory capacity only [10]. Since we did not measure working memory capacity in the present study, we cannot rule out the possibility that there might be a moderating effect. Finally, previous studies used different stress induction protocols, mainly differing with respect to whether they included a physical (MAST, SECPT) in addition to the psychosocial stress component (MAST, SECPT, TSST). In the current study, we have employed two different stress induction protocols so we can conclude that our results do not hinge on the specific stress induction protocol.

To summarize, our study lends further support to a growing number of recent studies showing no general shift towards more habitual (approach or avoidance) behavior following a short phase of acute stress [4,16]. Moreover, we show that different types of goal-directed behavior, here, learning novel action-outcome contingencies and choosing actions in order to produce certain outcomes, remained fully intact after stress both in terms of accuracy and response speed. Based on the present data alone we cannot decide whether this reflects a general resilience, which might be related to higher working memory capacity, or a certain age span, or is the result of an active compensating mechanism, which might not be available in other populations or after more prolonged exposure to a stressor. Furthermore, glucose administration prior to the experiment aimed at keeping glucose levels constant across participants and ensuring a robust cortisol response to the stressor. While we are not aware of any studies that have previously associated glucose levels with the balance between goal-directed and habitual behavior, glucose has been shown to affect cognitive processes, in particular facilitating memory [32,33], and thus could have potentially contributed to high performance in the task. Using the habit-goal competition paradigm [17], we have previously shown that the individual behavioral habit impact was predicted by a decreasing involvement of brain regions involved in goal-directed action control, such as the angular gyrus of the parietal cortex, rather than by an increasing of involvement of brain regions in habitual action control, such as the posterior putamen, during the training phase when habitual action tendencies were induced. This pattern of results has recently been corroborated by another study [44]. Thus, under circumstances that additionally tax the goal-directed system, stress might allow habitual action tendencies to impact behavior more strongly.
